# Anti-D Alloimmunization Following Rhesus-Incompatible Platelet Transfusion in a Myelodysplastic Syndrome Patient

**DOI:** 10.7759/cureus.57165

**Published:** 2024-03-29

**Authors:** Rahimie Hanafi, Razan Hayati Zulkeflee, Mohd Nazri Hassan, Nur Ilyia Syazwani Saidin, Sumaiyah Adzahar, Rosline Hassan

**Affiliations:** 1 Department of Ophthalmology and Visual Science, School of Medical Sciences, Universiti Sains Malaysia, Kota Bharu, MYS; 2 Faculty of Medicine, Universiti Sultan Zainal Abidin (UniSZA), Kuala Terengganu, MYS; 3 Department of Hematology, School of Medical Sciences, Universiti Sains Malaysia, Kota Bharu, MYS; 4 Department of Pathology, Faculty of Medicine, Universiti Sultan Zainal Abidin (UniSZA), Kuala Terengganu, MYS

**Keywords:** rhd-negative, rh immune globulin, anti-d, alloimmunization, myelodysplastic syndrome

## Abstract

Patients with myelodysplastic syndrome (MDS) often need platelet transfusions to address thrombocytopenia. The risk of alloimmunization, particularly in Rhesus (Rh) incompatibility between donors and recipients during platelet transfusions, is heightened, especially with whole blood-derived pooled platelets as opposed to apheresis platelets. Although the occurrence of alloimmunization from platelet transfusions is minimal, there is an ongoing debate about whether Rh immune globulin (RhIg) should be administered to Rhesus D (RhD)-negative recipients of RhD-positive platelet units. We present a unique case of anti-D alloimmunization in a 56-year-old patient with underlying MDS following multiple platelet transfusions but never received packed cell transfusion or anti-D immunoglobulin. Some studies advocate for RhIg administration in specific scenarios and for certain patient populations. This case underscores the importance of considering Rhesus compatibility or administering anti-D immunoglobulin in cases where frequent platelet transfusions are required.

## Introduction

Myelodysplastic syndrome (MDS) is a clonal hematopoietic disorder and is defined as ineffective hematopoiesis resulting in (i) cytopenias, (ii) peripheral blood or bone marrow dysplasia, and (iii) clonal cytogenetic abnormalities. One of the mainstay therapies for MDS is red cell or prophylactic platelet concentrate (PC) transfusion for severe symptomatic anemia or thrombocytopenia, respectively, to reduce the risk of bleeding [1].

Under typical circumstances, PC transfusions usually only require compatibility with the ABO blood group, disregarding the Rhesus D (RhD) status. This is because the RhD antigen, the most immunogenic one, is exclusively present in red blood cells (RBCs) and not in platelet cells. However, in specific situations, particularly for individuals of childbearing potential who are RhD-negative, a single anti-D prophylaxis dose of 300 μg is deemed necessary, which is outlined by the British Committee for Standards in Haematology (BCSH) guideline from 2014. This is to prevent alloimmunization after receiving two doses of RhD-incompatible platelet transfusions within a two- to four-week timeframe [2].

Platelet concentrates can potentially have RBC contamination, which is influenced by the platelet preparation method. In the context of whole blood donation, each platelet concentrate typically contains <1 × 10^9 ^(<0.1 mL) (range 0.036-0.59) mL RBCs. Consequently, there exists a risk of anti-D alloimmunization when RhD-positive platelets are administered to RhD-negative patients [3]. An alternative method, processing platelet concentrates through apheresis from a single donor, may reduce RBC contaminations. Each apheresis product contains fewer RBCs, ranging from 0.00017 to 0.009 mL, than those obtained through whole blood donation. As a result, cross-matching is not necessary in this apheresis-based process [4].

This case underscores an instance of anti-D alloimmunization arising from frequent platelet transfusions in a Malay male with an underlying myelodysplastic syndrome. In our institution, anti-D prophylaxis is not routinely administered during platelet incompatibility transfusions, including children and women of childbearing age.

## Case presentation

A 56-year-old male of Malay ethnicity, diagnosed with myelodysplastic syndrome with single lineage involvement, has been consistently receiving platelet transfusions for severe thrombocytopenia over the past two years. His hemoglobin levels have consistently remained within the normal reference range despite not having received packed red blood cell transfusions. During each hospital admission, he has faced recurring bleeding episodes, leading to the administration of over 90 units of platelets, including both whole blood platelet concentrates and single-donor platelets, all of which are RhD-positive. Notably, there is no record of prior rhesus immunoglobulin administration.

In his current admission, the patient experienced a nosebleed during a clinic visit, revealing a platelet level of 7 × 10^9^/L, necessitating a four-unit platelet transfusion. A request for blood group screening revealed a B RhD negative blood group with positive antibody screening. Further antibody identification identified anti-D with a titer of 1:256. The patient's rhesus phenotype was r’r, and the direct Coombs test was negative, excluding the possibility of an acute hemolytic transfusion reaction.

Immunohematology findings during the hospitalization are outlined in Table 1. Upon further investigation, it was discovered that the patient had never received packed red cell transfusions, only multiple single-donor platelets and random platelet concentrates. A single-donor unit of RhD-positive platelets (~250 cc) was administered without any complaints from the patient. Subsequent platelet counts after transfusion were 20 × 10^9^/L, with no further nosebleeds. The patient was discharged in good health and continued monthly follow-ups, with an increased dose of Eltrombopag olamine, maintaining a platelet count of more than 50 × 10^9^/L. This case highlights that the patient has experienced RhD alloimmunization, and the likely cause is attributed to his history of platelet transfusions.

**Table 1:** Summary of immunohematology investigations of the presented case

ABO/RhD blood type (DiaClon, Bio-Rad, Switzerland)	B-negative
Antibody screening (ID-Diacell, Bio-Rad)	Positive
Antibody identification (ID-DiaPanel, Bio-Rad)	Anti-D
Antibody titer	1:256
Rh phenotype/genotype Rhesus gel card (DiaClon, Bio-Rad)	C+,E-,c+,e+ (r’r)
Direct antiglobulin test	Negative

## Discussion

During blood transfusion therapy, the occurrence of antigenic mismatch between donors and recipients can pose risks by triggering the production of antibodies against one or more erythrocyte antigens, leading to alloimmunization [5].

In the absence of the RhD antigen on platelet surfaces, the risk of anti-D alloimmunization is generally low. However, this risk increases if there are remaining RhD-positive red blood cells during platelet preparation, posing a potential threat of RhD alloimmunization in RhD-negative individuals. Instances of RhD alloimmunization have been documented in recipients even with as little as 30-50 µL of red blood cells [6]. Nevertheless, the practice of transfusing platelets based on Rh compatibility is not implemented due to logistical constraints and limited resources associated with Rh-negative platelets.

Anti-D antibody development depends on several factors. Immune status is a critical factor, with studies on immunosuppressed patients consistently showing a higher likelihood of lacking anti-D immunization. Additionally, the presence of residual RBC content in platelet products is a significant consideration. Some literature suggests that individuals receiving single-donor apheresis platelets may not necessarily need anti-D prophylaxis due to minimal RBC contamination.

In the aftermath of this case, the patient did not receive RhIg during his platelet transfusions despite the rhesus incompatibility. However, as the patient is male and exclusively receiving platelet transfusions, certain centers recommend the use of platelets without considering the RhD status and without administering RhIg prophylaxis. It is worth noting that RhIg has a half-life of three weeks and a 250 IU dose of anti-D immunoglobulin is typically deemed adequate to cover up to five adult therapeutic doses of RhD-positive platelets administered within a six-week timeframe [2].

This case report indicates a notable risk of anti-D alloimmunization due to residual RBC content in the platelet product given to an immunocompromised patient. The complications linked to anti-D alloimmunization may manifest as either an acute hemolytic transfusion reaction or serological problems.

This becomes particularly pertinent when patients receive packed cells with rhesus incompatibility. Moreover, the presence of clinically significant anti-D antibodies can lead to delays in transfusions, further complicating the identification of compatible blood. Table 2 tabulates a summary of clinical and laboratory findings recorded in published instances of alloimmunization arising from plasma transfusion [7-9]. McLeod et al.'s research indicated that immunosuppressed patients had the highest occurrence of anti-D alloimmunization after receiving D-incompatible platelet transfusions, while in immunocompetent individuals, the production of anti-D typically occurred within approximately 1-5 months [10].

**Table 2:** Overview of clinical and laboratory observations documented in published cases of alloimmunization resulting from platelet and FFP transfusions RDP: random donor platelet, RhD+: Rhesus D-positive, AML: acute myeloid leukemia, FFP: fresh frozen plasma

Patient	20-year-old, male	21-year-old, male	56-year-old, female	72-year-old, male
Diagnosis	Dengue fever	Dengue fever	Subarachnoid hemorrhage and arachnoid cyst	AML with myelodysplasia-related changes
Patient's blood group	O RhD-negative	O RhD-negative	A-negative	B RhD-negative
Type of plasma transfusion received	4 units of RDP (2RhD+)	16 units of RDP (10 RhD+)	Apheresis RhD+ FFP and RhD- RBC	Random donor platelet transfusion
Antibody identification	Anti-D	Anti-D and anti-C	Anti-D 1:64	Anti-D, anti-C, and anti-E
Reference	[7]		[8]	[9]

Several mitigation strategies can be employed to decrease anti-D alloimmunization. One approach involves offering apheresis platelet transfusion, which has minimal red blood cell contamination. Another strategy entails administering RhIg before transfusing RhD-positive platelets [11].

## Conclusions

We aim to draw attention to our experiences to fellow professionals' enhanced management of platelet incompatibility transfusions to mitigate the risk of anti-D alloimmunization. This is critical not only for individuals of childbearing age but also especially pertinent for those anticipating frequent future blood transfusions. Despite its rarity, we recommend heightened vigilance in the blood bank regarding Rhesus compatibility during platelet transfusions and regular monitoring of erythrocyte contamination levels in platelet concentrates.
